# Retrospection of heatwave and heat index

**DOI:** 10.1007/s00704-021-03854-z

**Published:** 2021-11-11

**Authors:** Amit Awasthi, Kirti Vishwakarma, Kanhu Charan Pattnayak

**Affiliations:** 1 Department of Applied Sciences, University of Petroleum & Energy Studies, Dehradun, 248007 Uttarakhand India; 2 Department of Aerospace Engineering, University of Petroleum & Energy Studies, Dehradun, Uttarakhand India; 3 School of Earth and Environment, University of Leeds, Leeds, UK

**Keywords:** Climate change, ERA5, Extreme events, Heat wave, Heat index

## Abstract

The frequency and intensity of extreme events especially heat waves (HW) are growing all around the world which ultimately poses a serious threat to the health of individuals. To quantify the effects of extreme temperature, appropriate information, and the importance of HW and heat index (HI) are carefully discussed for different parts of the world. Varied definitions of the HW and HI formula proposed and used by different countries are carried out systematically continent-wise. Different studies highlighted the number of definitions of HW; however, mostly used Steadman’s formulae, which was developed in the late 1970s, for the calculation of HI that uses surface air temperature and relative humidity as climatic fields. Since then, dramatic changes in climatic conditions have been observed as evident from the ERA5 datasets which need to be addressed; likewise, the definition of HW, which is modified by the researchers as per the geographic conditions. It is evident from the ERA5 data that the temperature has increased by 1–2 °C as compared to the 1980s. There is a threefold increase in the number of heatwave days over most of the continents in the last 40 years. This study will help the researcher community to understand the importance of HW and HI. Furthermore, it opens the scope to develop an equation based on the present scenario keeping in mind the basics of an index as considered by Steadman.

## Introduction

The occurrence and strength of extreme heat events are increasing all around the world which has direct and indirect impacts on the health of living beings and the ecological system. Extreme heat events are physical hazards that cause health issues in eerie (Kent et al. 2014). These events also affect the earth’s warming process. One of the main reasons for the warming of the earth is due to the increase in concentrations of greenhouse gases received from different anthropogenic sources that produce a higher level of pollutions (Fischer et al. 2004). Researchers believe that this scenario of increase in temperature (Pattnayak et al. 2017) will continue aggressively in the future as well if no necessary steps are taken to control the pollution level. Modernization and industrialization provide new technologies that improve the lives of human beings, but along with this, severe impacts are observed on the environment. These new technologies consume a lot of energy that later on leads to produce air pollution and a lot of heat in the atmosphere which triggers many respiratory diseases (Chen et al. 2021; Fischer et al. 2004; Luan et al. 2019). As a result, the average temperature is increasing day by day which increases the possibilities of severe heatwave events that later on are responsible for the mortality rate (Hu et al. 2019). Climate change is considered to be the foremost reason behind these extreme events happening aggressively all around the world (Frich et al. 2002; Pattnayak et al. 2019; Trájer et al. 2019). The health impacts of heatwaves typically start from heat cramps, heat exhaustion, dehydration, and heatstroke that later on may lead to death, if necessary, care is not taken. Hence, along with other natural and anthropogenic reasons (Abbasnejad et al. 2019; Agarwal et al. 2010, 2013, 2014; Awasthi et al. 2017; Panda et al. 2020), extreme heat events also play an important reason for the number of causalities throughout the world (Fischer et al. 2004; Wang and Yan 2021).

The impact of the heatwave (HW) is not limited to the health of human beings (Kotharkar and Ghosh 2021; Nitschke et al. 2011; Pascal et al. 2021; Zhang et al. 2017) but agriculture, ecosystems, and the national economy too are also significantly affected by it (Luan et al. 2019). Extreme events like HW, cold waves, drought, floods, cyclones, tornadoes, hurricanes, etc. initially gained attention in the developed countries but, soon due to its global impacts it is discussed and of keen interest worldwide. However, it must be noticed that the events such as floods and tornadoes are a matter of concern if their occurrence leads to loss of human life, but events such as heatwaves and cold waves have both direct and indirect long- and short-term impacts on the environment and human life.

Among all the months, these extreme heat events normally occur in mid-summer and less intense heat waves also occur in early autumn and during spring. In summers, the value of feel like the temperature is much higher than that of the actual temperature. The feel-like temperature is scientifically termed as the Heat Index (HI) which accounts for humidity along with the actual temperature (Montero et al. 2013). Humidity is the amount of moisture or water vapors present in the atmosphere which is expressed in the terms of Relative Humidity. It makes the hot temperature even more unbearable as the presence of humidity in the environment reduces the ability of the body to cool itself. Due to this, in most HI formulas, the contribution of both, air temperature and relative humidity is visible.

There is no globally accepted standard HW definition (discuss in detail in coming section), although it is commonly defined as limited successive days with high temperatures above a certain threshold based on community and physiology (Montero et al. 2013). To study and manage heat-related risks imposed on health, HW and HI are needed to be considered wisely. The extent of adverse effects of HW can be carefully studied if HI is known for a particular region that has a direct relation with HW. Hence, in the coming sections, the definition of HW and the concept of HI are studied to understand their basics. The aim of the assessment done in the present paper is to clarify the definition of HW and HI so that their understanding shall help to reproduce the same. The key purpose is to understand the methods used by different countries to develop the relation of HI for their country as characterized by the data accumulated from different sources. In this paper, efforts are made to systemize maximum information about the basics of HW and HI to build a foundation for its understanding and to open new perspectives for young researchers, environmentalists, and policymakers, etc. for further modification and its application to minimize the hazardous effects of HW. The following section deals with the descriptions of HW and HI. Section 3 deals with the datasets used and the methodology adopted for this study. The classification of HW and HI across the different regions of the world has been discussed in Sect. 4, while the discussion has been made in Sect. 5. Summary and concluding remarks are provided in Sect. 6.

## Descriptions of heat wave and heat index

### Heat wave (HW)

Extreme heat events are named like HW, heat advisory, excessive heat event, and hot spell, etc. are commonly referred to as temperatures that are either unusually high compared to characteristic local environments or extend to the level which may harm human health and infrastructure. HW is one of the slightest considered terms which have systematic significant risks to the world. HW is tough to characterize, and there is no specific meaning of an HW because similar meteorological situations can establish an HW in one place but not in another. Definition of HW is based on the three categories i.e., media, area-wise, and an all-inclusive one (Perkins and Alexander 2013). Due to the absence of a generic definition of HW, different countries are using different criteria for HW, which are observed from Table 1 (Tong et al. 2010). It demonstrates the diverse definitions used to characterize HW and distinctive criteria were taken to characterize its meaning in the diverse era. To characterize the heat waves from various nations, distinctive sources are taken. It is clearly understood from Table 1 that different nations used their definition in which reference temperature and duration varied as per the variation in their meteorological parameters.

**Table 1:** Definitions of heat wave used in literature (Tong et al. 2010)

Definition	Region	Reference
If a minimum temperature not below 26.7 °C is recorded for at least 48 consecutive hours, it is termed as heat wave	USA	(Robinson 2001)
If the daily maximum temperature exceeds 35 °C for a period of 3 or more consecutive days, it is termed as heat wave	Australia	(Hansen et al. 2008)
If the daily maximum temperature exceeds 37 °C for a continuous period of 2 days, it is termed as heat wave	Global definition	Global Climate Report 2013(NOAA 2013)
Periods of at least 3 consecutive days when the maximum and the minimum temperature, averaged, were simultaneously greater than their respective 95^th^ percentile	France	(Rey et al. 2007)
For Global analysis except for larger areas like Africa and S. America, if the daily maximum temperature exceeds average temperature by 5 °C, it is termed as heat wave	Globe except for larger areas like Africa and S. America	(Frich et al. 2002)
For Europe, China, Russia, Eastern US, Chicago, if the daily maximum temperature exceeds 35 °C for at least 2 consecutive days, it is termed as heat wave	Europe, China, Russia, Eastern USA, Chicago	(Russo et al. 2017)
If the daily maximum temperature exceeds 33.59 °C for a continuous period of 3 days, it is termed as heat wave	Global definition	Global Climate Report 2015(NOAA 2016)
If the daily maximum temperature exceeds 32.65˚C for a continuous period of 3 days, it is termed as heat wave	Global definition	Global Climate Report 2015(NOAA 2016)
Heatwaves were defined when daily maximum temperature values exceeded the 90th percentile for at least 3 consecutive days	South Africa	(Lyon 2009)

Table 1 highlighted that there are several diverse definitions of HW which are adapted by the researchers based on their local climatic zones. These HW definitions are based on the duration of HW, a threshold of temperature (e.g., a relative threshold or an absolute threshold), and temperature indicator e.g., daily average, minimum, and maximum temperature, etc. To support the above statement, a review done by the various authors on different continents will be highlighted in the later section with the only aim that is to understand the logic behind the adoption of a different definition of HW. Before that, first of all understand briefly about the HI.

### Heat index (HI)

Along with temperature, relative humidity (RH) plays a vital role to calculate the effects of extreme heat events, because it is not only the heat that poses an effect. It is also the RH that is used to determine how hot and humid it feels based on the combined effect of temperature and humidity. This combined effect is represented by the mathematical term called HI. Basically, HI measures how hot it feels actually when RH is considered with air temperature. HI is one of the methods which can be used to access the potential risk of an extreme event like HW. As the environmental conditions of all the countries in the world are not the same, different countries use different definitions to define the HW based on different metrological parameters range. Additionally, they calculated HI for their area using the Steadman Scheme. This is developed by using multiple regressions such as the Poisson Regression analysis technique on the meteorological data (Steadman 1979). This formula which is called HI is applicable over a certain threshold value of temperature depending on the maximum temperature and relative humidity and is shown in the equation below.

1 $$HI=-42.379+2.04901523xT+10.1433127xRH-0.22475541xTxRH-6.83783x{10}^{-2}x{T}^{2}-5.481717 {x10}^{-2}x{RH}^{2}+1.22874 {10}^{-3}x{T}^{2}xRH + 8.5282 {10}^{-4}xTx{RH}^{2}- 1.99x{10}^{-6}x{T}^{2}x{RH}^{2}$$

where HI: Heat Index in °F; RH: Relative Humidity in %, T: Ambient Dry Bulb Temperature in °F.

This formula is used by different researchers around different parts of the world to access the effects of HW on human beings. Based on HI, the heat index calculator or heat index chart provide different zones like caution, extreme caution, danger, and the extreme danger zone (Table 2).

**Table 2:** Risk conditions experienced by individuals at a different range of heat indices

Heat index	Risk conditions by prolonged physical activity
< 27 °C	Comfortable
27–32 °C	Fatigue
32–41 °C	Muscle cramps, sunstroke, heat cramps
41–54 °C	Sunstroke, muscle cramps, exhaustion, heatstroke
< 54 °C	Sunstroke/heatstroke

By understanding these zones, necessary steps will be taken to minimize the effects of HW. It is interesting to find in the coming section that most countries use Eq. (1) for the measurement of HI with their own modified HW definitions. HW studies and reviews done by authors in most of the continents of the world like the USA, Africa, Europe, Australia, and Asian countries like India, Taiwan, and Bangladesh, are discussed in the coming section of this paper. Studies are selected in such a way that it gives the basic idea of HW and HI and covers almost all the continents. Definitions used to characterize the HW of these countries are summarized in Table 3 which are elaborated in the next sections, along with several other countries to understand the reason behind the difference in the definitions.

**Table 3:** Definitions of heat wave discussed in the present paper

Definition	Days		Region	Reference
“A time interval of at least 2 days with maximum apparent temperature exceeding the 90^th^ percentile of the monthly distribution.”	2	Europe		(D’Ippoliti et al. 2010)
“If the daily maximum temperature exceeds 30 °C for a continuous period of 3 days, it is termed as heat wave in Hilly regions of India.”	3	Hilly Regions, India		(Deoras 2016)
“If the daily maximum temperature exceeds 40 °C for a continuous period of 3 days, it is termed as heat wave in plain regions of India.”	3	Plain Regions, India		(Deoras 2016)
“If the daily max temperature exceeds 37 °C for a continuous period of 3 days, it termed as heat wave in coastal regions of India.”	3	Coastal Regions, India		(Deoras 2016)
“Period ≥ 3 consecutive days with maximum temperature above the 90th percentile of daily maxima temperature, centered on a 31-day window.”	3	Africa		(Ceccherini et al. 2017)
“The top 5% ($32.65uC) of daily maximum temperatures for a continuous 5 days period.”	5	Brisbrane, Australia		(Tong et al. 2010)
“Absence of normal pre-monsoonal rainfall which is brought by aberrant strong low-level westerly winds and weak southerlies for consecutive 10 days is defined as heatwaves.”	10	Bangladesh		(Nissan et al. 2017)
“If the sweltering climate continues for 16 successive days, with temperatures achieving 36 °C, it is termed as heatwave.”	16	Taiwan		(Lin et al. 2013)

## Data and methodology

Weather variables were used in this study as direct input to the HI equation. Data were obtained from a public dataset, ERA5 (Hersbach et al. 2020), generated and hosted by the European Centre for Medium-range Weather Forecasts (ECMWF). ERA5 is a reanalysis dataset (hereafter called proxy observed) that provides weather variables homogeneously distributed at the global scale (30-km horizontal resolution), which are obtained from point-specific ground, ocean, atmosphere, and satellite observations through the application of a data assimilation system based on the ECMWF Integrated Forecasting System and a 4-dimensional variational analysis (4D-Var). In this study, the following surface variables were retrieved for the European domain as a proxy for meteorological observations: the daily mean temperature at 2 m and relative humidity. Both variables have a 3-h time resolution. The study period has been chosen as 40 years starting from 1979 till 2018, to understand the mean behavior of these meteorological parameters and their trends over different parts of the globe. The time series over the specific regions have been extracted by taking the mask of those specific regions. This daily data series is used to prepare the mean annual cycle or the temporal variation of the climatic fields over different regions.

## Worldwide classification of heatwave and heat index

**Fig. 1:**
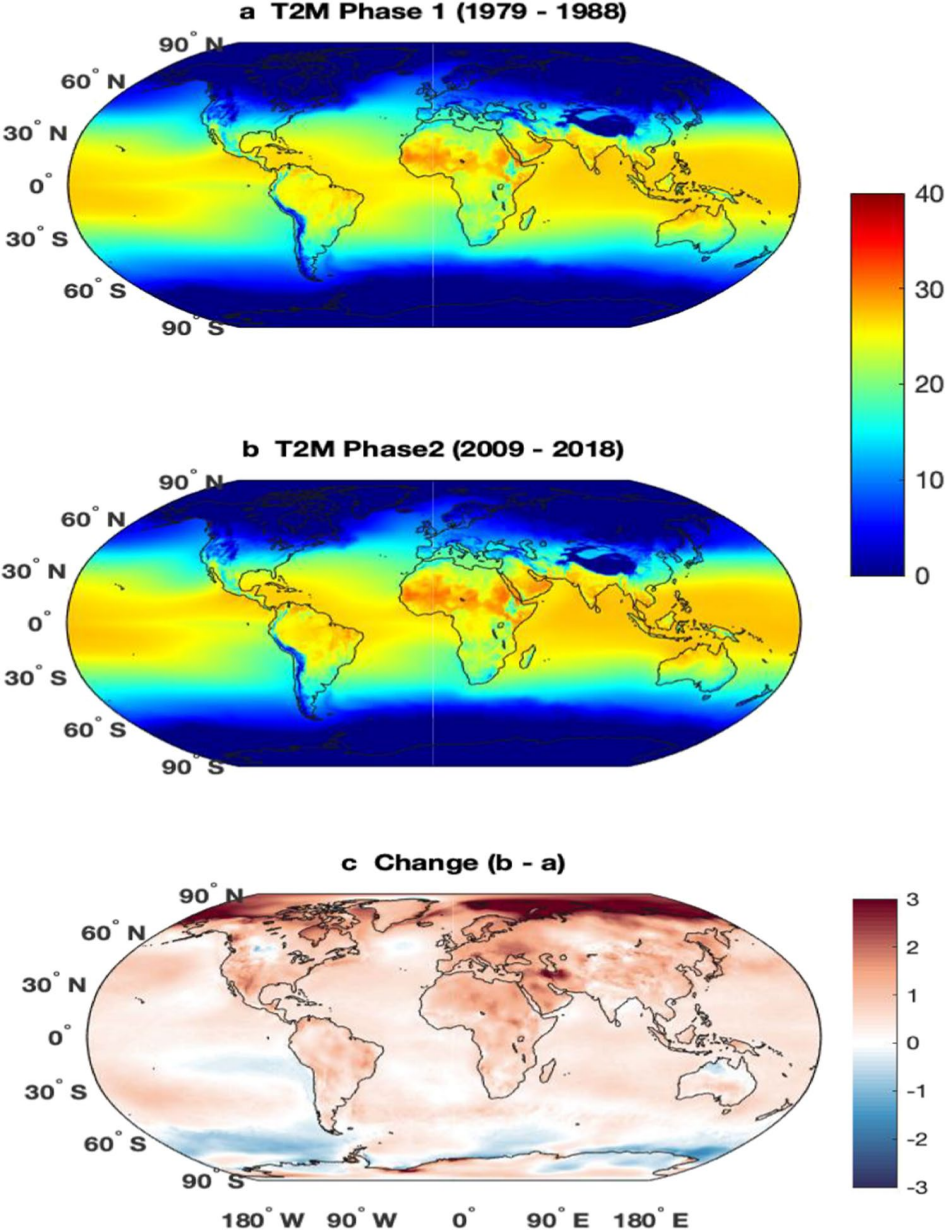
Mean temperature at 2 m (°C) from ERA5 for the period (**a**) 1979–1988, (**b**) 2009–2018, and (**c**) change between these two periods

**Fig. 2:**
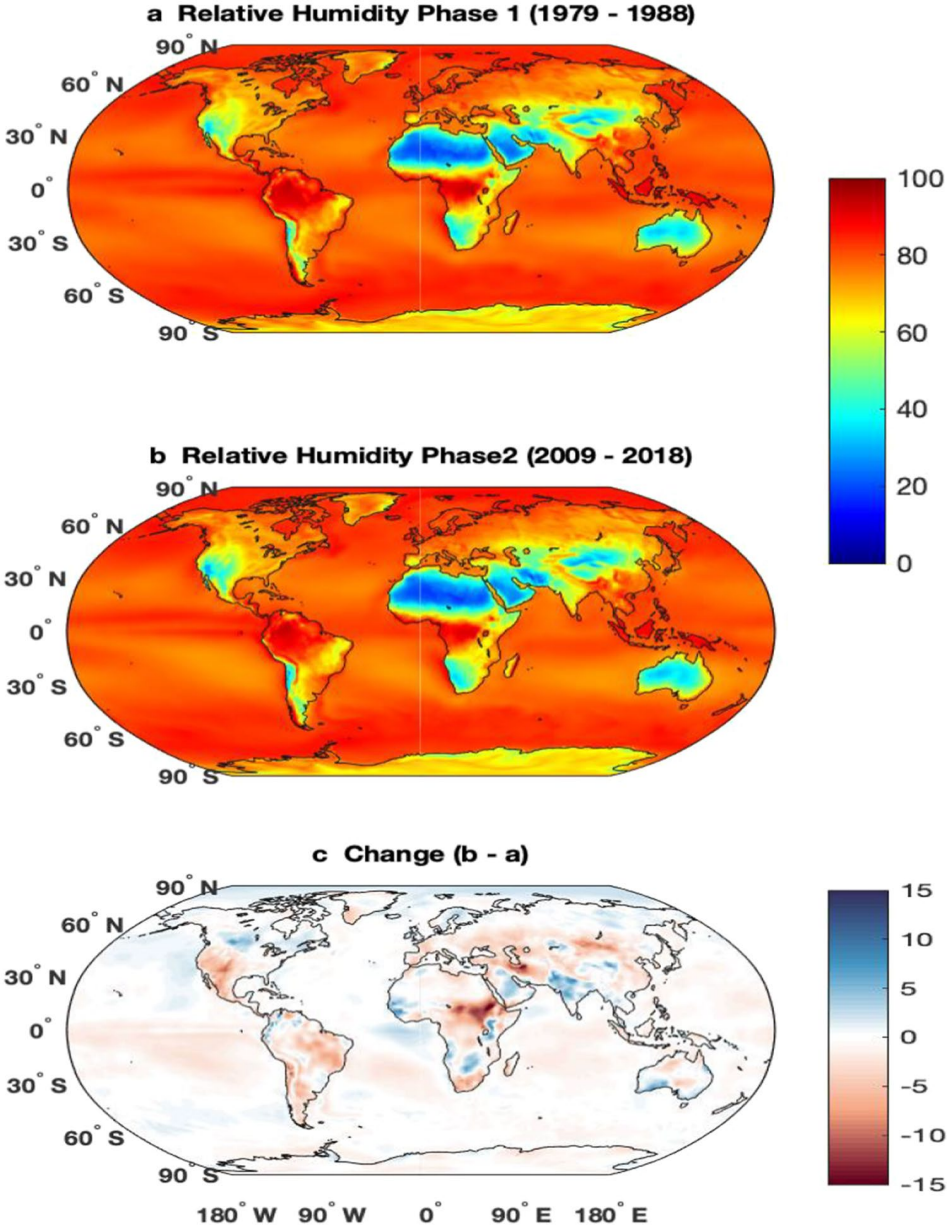
Mean relative humidity (%) from ERA5 for the period (**a**) 1979–1988, (**b**) 2009–2018, and (**c**) change between these two periods

HW and HI have been used differently in different parts of the world. The definition mainly differs because of the temperature and relative humidity of that region. Many studies suggest that the global temperature has been increased more rapidly in the last decade and therefore the RH has also changed significantly (Byrne and O’Gorman 2018; Cheng et al. 2018; Lau et al. 2021; Liu et al. 2018; Pattnayak et al. 2016; Sharma and Babel 2014; Tiwari et al. 2016). Relation between temperature and RH and their effect on health issues is studied by different authors (Awasthi et al. 2020; Mozumder et al. 2021; Sahu et al. 2021; Talbot et al. 2021). To examine, how the temperature and RH changes have affected the HI, the study period has been divided into two periods which are 1979 to 1988 (1980s) and 2009 to 2018 (2010s). Figure 1 depicts the mean air temperature at 2 m and Fig. 2 shows the RH for the two periods and changes in the 2010s with respect to the 1980s. It can be observed that the temperature increased (Fig. 1) over most of the globe except over a few parts of Antarctica (Doran et al. 2002; Oliva et al. 2017; Sancho et al. 2017) and the eastern part of the equatorial Pacific ocean (Kosaka and Xie 2013; Martínez-Garcia et al. 2010; Moum et al. 2013; Zhang et al. 2010). The rate of increase in temperature is more in the polar region than in the tropical and subtropical regions. In the northern pole, the temperature has increased by more than 3 °C, while the temperature change is about 1–2 °C over the tropical region. The change in RH (Fig. 2c) is a more or less similar pattern to the temperature (Fig. 1c). In most of the regions, where the temperature has increased, the RH has also increased and vice versa. In the Antarctica region, the RH decreased and over the Arctic region, it is increased. Furthermore, the change in the climatic fields over the continents has been discussed in the following subsections. Few regions have been selected in each of the continents just to show how the climatic fields have changed and how it may affect HI.

### Asia

Asia is one of the largest and populated continents of the world and is supposed that 60% of the total population of Earth is living here. Asia has extremely diverse climates range from arctic and subarctic in Siberia to tropical in southern India and Southeast Asia. Some of the countries from Asia are discussed in the incoming section, where it is understood that with the vast variation in temperature across Asia mostly used the same Eq. 1 for HI with a different definition of HW.

#### China

**Fig. 3:**
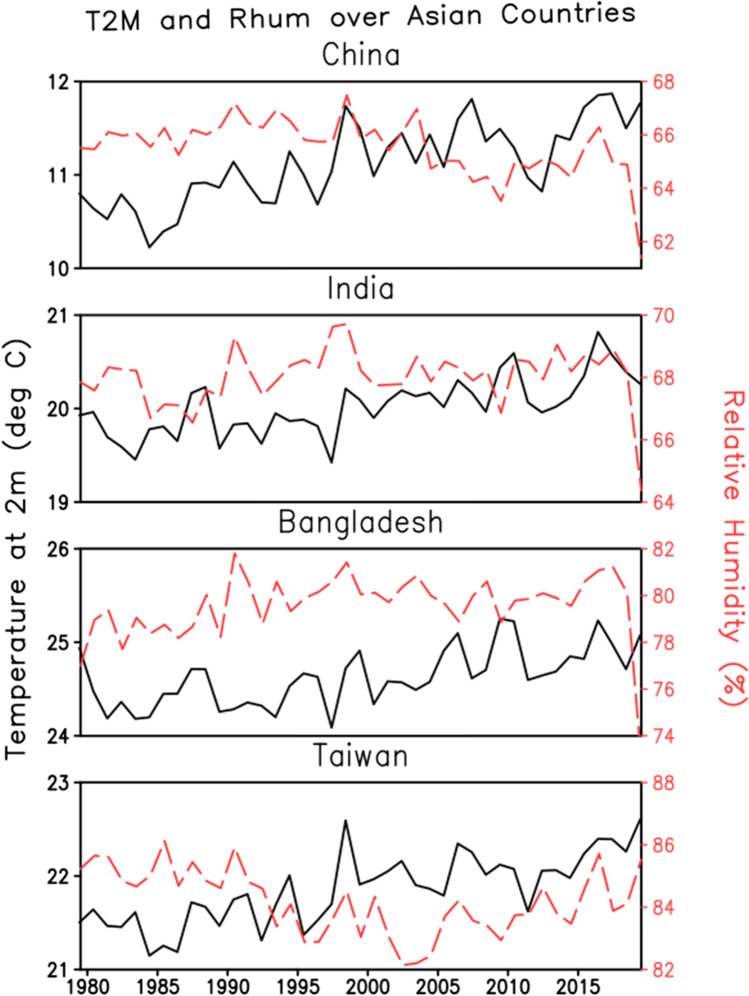
Interannual variation of temperature (°C) and relative humidity (%) over Asian countries for the period 1979 to 2018 from ERA5. The climate fields are averaged over China (top panel), India (2^nd^ panel from the top), Bangladesh (3^rd^ panel from the top) and Taiwan (bottom panel)

**Fig. 4:**
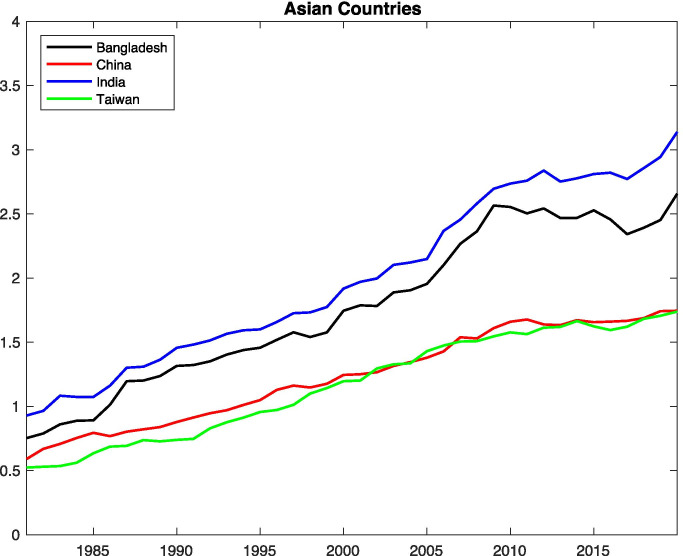
Trend of heatwave days (no. of days per year) over the Asian countries for the period 1979 to 2018 from ERA5

Ding et al. (2010) summarize the details of HW in China during 1961–2007 (Ding et al. 2010). Two definitions are quoted in this paper, one definition is based on absolute criteria when the daily maximum temperature is greater than 35 °C. Another definition is relative, i.e., if the daily maximum temperature is greater than the 90^th^ percentile threshold of the local daily temperature distribution of the data. The analysis shows that HW events sharply increased in northwestern China and eastern China. The frequency and intensity of HW both increase significantly in China. ERA5 temperature suggests that the average temperature has increased by about 2 °C (Fig. 3a) and the number of heatwave days has increased 3 times (Fig. 4) in the last 40 years.

#### India

India has a wide range of temperatures. States lying in the Eastern part of India have comparatively high temperature than western states but lower than that of northern states. Southern states like Tamil Nadu, Kerala have a humidified temperature throughout the year. According to National Survey, over 500 people die in a 3-day time period from May 20 to May 22, 2010. In May 2010, Ahmedabad that is lying in the western part of India faced HW where the temperature exceeded 46.8 °C. A study was done to quantify the effect of HW on the mortality rate and to demonstrate the HW impact and calculate the correlation with mortality. Till 2009, the daily average maximum temperature observed by the Ahmedabad was 40˚C, but an average temperature over 45˚C was faced in May 2010 which resulted in a huge loss of lives (Azhar et al. 2014; Knowlton et al. 2014). In May 2015, Indian cities were struck by extreme warm waves, which regularly keep going during the dry season i.e. from March to July with the highest temperatures in April and May. It is documented that up to 3^rd^ June 2015, 2500 individuals died at different locations in India (Rohini et al. 2016). According to Indian Meteorological Department (IMD), HW is considered for plains, when the temperature exceeds 40 ˚C, and for Hilly areas, when the temperature exceeds 30 ˚C. If the extreme temperature stays at least 45˚C regardless of the ordinary greatest temperature, it is termed as a heatwave. However, this is an average value that may differ for different regions. The majority of countries use the Steadman scheme to calculate the HI for their region. In the Himalaya regions of India, a different approach was used to calculate the HI i.e., Tree Rings method. Since there is a large variation of temperature in the Himalayan Region, so measurement of HI for the long term is a tedious process. Researchers chose generally two types of trees found in the Himalayan region i.e., Cedrusdeodara and Pinus roxburghii for these types of measurements. With the study of tree-ring width, Rainfall (mm), Moisture Content, and Temperature are known. By using tree ring methods, Ram and Borgankar (2016) measured the different values of HI for different months in the Himalayan Region regarding the different values of average temperature which are calculated on the basis of rainfall, moisture content, humidity, and temperature**.** They have found that May and June are the most vulnerable times, as the HI and temperature have maximum values during this period. The change in the annual temperature is about 1 °C over India and there is no significant trend in relative humidity pattern (Fig. 3b). However, India has witnessed a threefold increase in the number of heatwave days (Fig. 4) in the last 40 years. Our findings over India agrees well with the study made by Mukherjee and Mishra (2018).

#### Bangladesh

Bangladesh faced one of the major heat waves between January 1994 and December 2002, as a total 13,720 lives were lost due to HW excluding external causes. In Bangladesh, absence of normal pre-monsoonal rainfall which is brought by aberrant strong low-level westerly winds and weak southerlies for consecutive 10 days is defined as heatwaves (Nissan et al. 2017). During the HW in 2008 for consecutive 8 days, at least 3800 lives were lost due to excess heat in which 2/3 of lives were of people above 65 which indicates that the elderly is especially affected by the HW. Between 1982 and 2008, a total of 49,426 children under the age of 5 lost their lives due to HW with an average of 153 deaths per 1000 live births per month. This data had 4725 girls and 5459 boys that died at an age less than 5 months which resulted in 23 deaths/1000 births in a month (Babalola et al. 2018).

The mean HI value of Bangladesh (42–50 °C) found matches to the average temperature of India during the summer season. The study of HI in Bangladesh was performed by dividing the country into four regions: (i) Central Area (CA), (ii) Northwest Area (NWA), (iii) Southwest Area (SWA), and (iv) East Area (EA) as shown in Table 3. This study by division of the country into various parts will help in understanding the procedure or methodology to develop HI for India. Using the real-time data of 20 stations out of 35 stations from Bangladesh Meteorological Department which included monthly dry bulb temperature and relative humidity for the period 1961–1990, HI was calculated. Such a formula in Eq. (1) is appropriate only when air temperature and humidity are higher than 26 °C and 39%, respectively. The HI value has an error of ± 1.3°F, as it has been obtained by multiple regression analysis (Steadman 1979). It can be observed from Fig. 3c that there is no significant change in both the climatic fields observed over Bangladesh. Our study shows that the number of HW (Fig. 4) has been increased by 2.5 times as compared to 1980s.

#### Taiwan

HW study was conducted in Taiwan, where extended mortality during warm waves has been attributed generally to cardiovascular conditions and cerebrovascular issues (Lin et al. 2013). Another study comprised of calculating the daily mean HI relationship with mortality by using Poisson regression analysis with generalized linear models (GLMs) in 6 major cities (Taipei, Keelung, Chiayi, Taichung, Kaohsiung, and Tainan) in Taiwan which lied between 26.4 °C (79.5 °F) and 28.6 °C (83.4 °F). The different parameters were studied, and average values were taken. The data was collected through Central Weather Bureau and US National Weather Service (Sung et al. 2013)**.** The meteorological data of Taiwan was used to calculate the value of HI from 1994 to 2008. It contained the least daily mean values of Temperature and RH. When the meteorological data was substituted in Eq. (1), it was found that Keelung had the least value of HI i.e., 26.4 ± 5.2 °C, and Kaohsiung had the greatest one, i.e., 28.6 ± 4.9 °C as they were dependent on the magnitude of Temperature and RH. Taiwan has experienced warming of about 1.5 °C in the last 40 years, however, the increase rate is more during the recent decades (Fig. 3d). The trend of HW over the country roughly follows the trend of China (Fig. 4).

### Africa

**Fig. 5:**
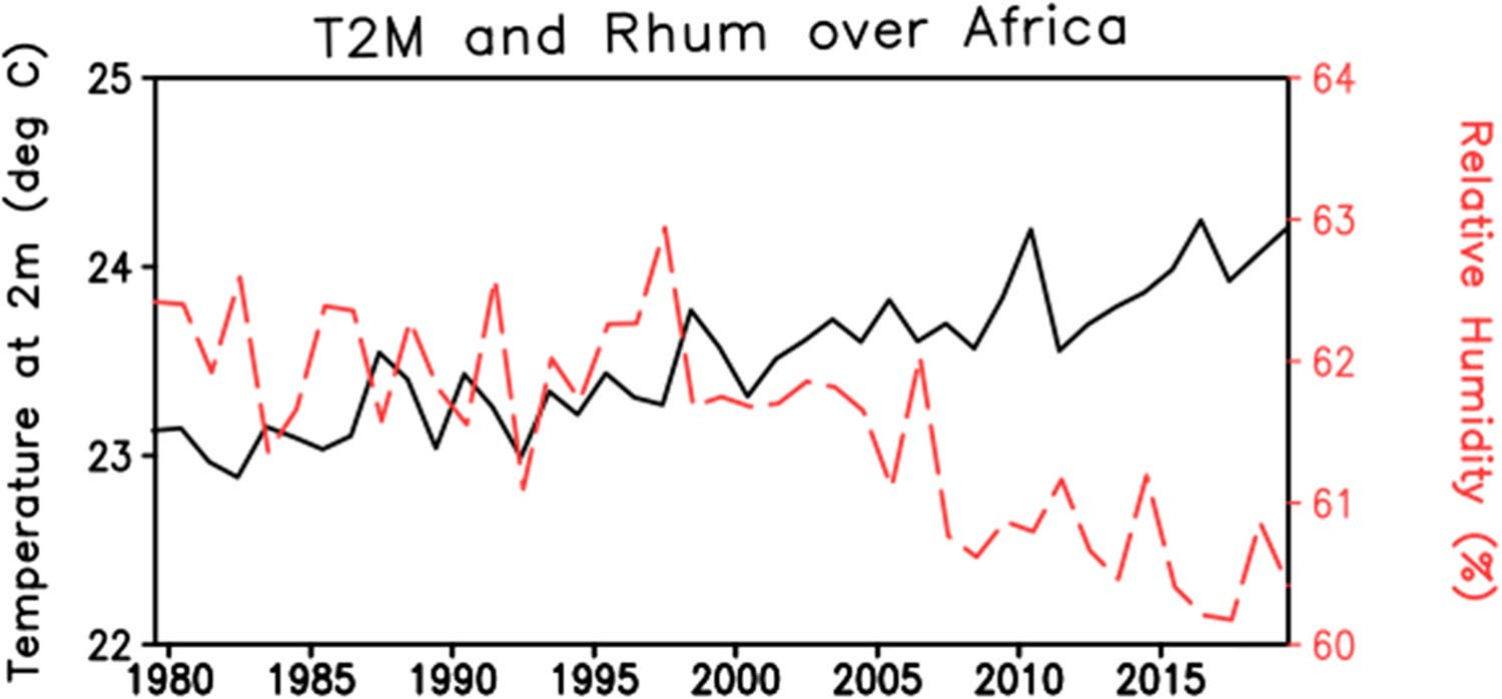
Interannual variation of temperature (°C) and relative humidity (%) over the African continent for the period 1979 to 2018 from ERA5. The climate fields are averaged over the whole African continent

One of the hottest continents of the World is Africa in which the maximum average temperature of Earth is quoted at Dallol, Ethiopia. Many severe HW events were observed in different parts of Africa. One of the studies done by Lyon 2009 in Southern Africa, defines HW as a daily maximum temperature greater than 95 percentiles for at least 3 consecutive days. Ceccherini et al. (2017) reviewed the HW in Africa for a period from 1981 to 2015, consider the HW as a “period ≥ 3 consecutive days with maximum temperature above the 90th percentile of daily maxima temperature, centered on a 31-day window” (Ceccherini et al. 2017). Definition of HW is designed or modified after considering the metrological conditions of African countries. Figure 4 shows the interannual variation of temperature and relative humidity for the last 40 years. It suggests that the temperature has increased by 1 °C and the relative humidity has decreased by about 3% (Fig. 5). After 2000, both the climatic fields have been changed significantly. According to ERA5 data, the heatwaves have increased by 3.5 times over the continent (Fig. 9).

## North America

**Fig. 6:**
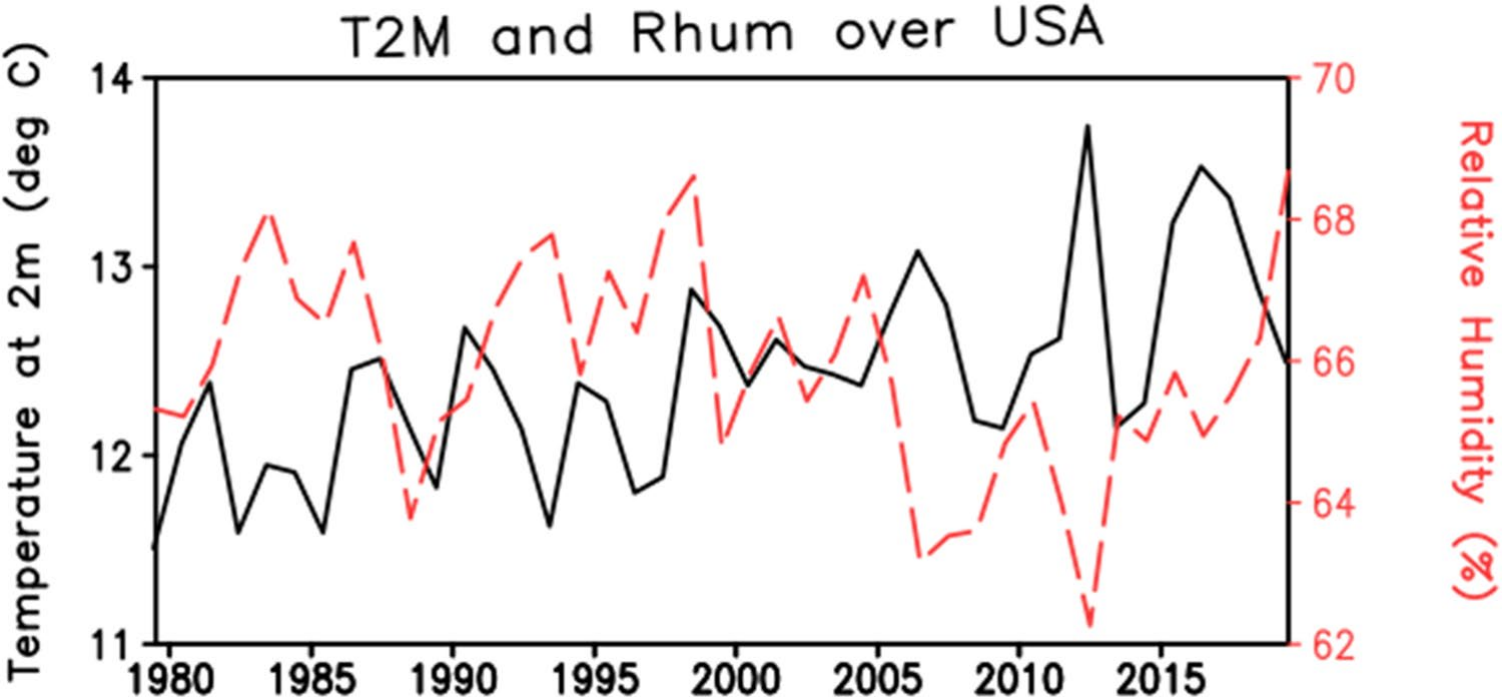
Interannual variation of temperature (°C) and relative humidity (%) over the USA for the period 1979 to 2018 from ERA5. The climate fields are averaged over the USA

The USA has been chosen to represent North America for this study, Smith et al. (2013) reviewed the HW in the USA for 40 years (Smith et al. 2013). In this paper, the authors’ summarize data from the North American HW indices from 1979 to 2011. Sixteen definitions of HW indices were quoted, which differ in terms of temperature type i.e., minimum, maximum or average, threshold, durations, and type i.e. relative and absolute. etc. It is observed from this paper that HW is not unique meaning, but it is changed as per the geographical conditions, but focus and basic objective are the same in all the studies. There is a significant increasing trend in temperature (Fig. 6) and HW (Fig. 9) have been observed in the last 40 years.

### Europe

Europe was affected by two of the worst heat waves in summer 2003, the extreme maximum temperature of 35 to 40 °C was frequently recorded in July, which extended in August in most of the southern, and central countries from Germany to Turkey. It led to the hottest summer ever recorded in Europe since 1540, with estimated excess mortality varying between 25,000 and 70,000 death in Western Europe (D’Ippoliti et al. 2010). A definition was given to HW according to which HW for Europe is defined as “A time interval of at least 2 days with maximum apparent temperature exceeding the 90^th^ percentile of the monthly distribution or a time interval of at least 2 days in which minimum temperature exceeds the 90^th^ percentile and maximum apparent temperature exceeds median monthly value”. Concerned with this treacherous HW, an attempt was made to develop the HI equation which could be used for further analysis in two regions of Europe, i.e., Marmara and Naples region.

#### Marmara Region

**Fig. 7:**
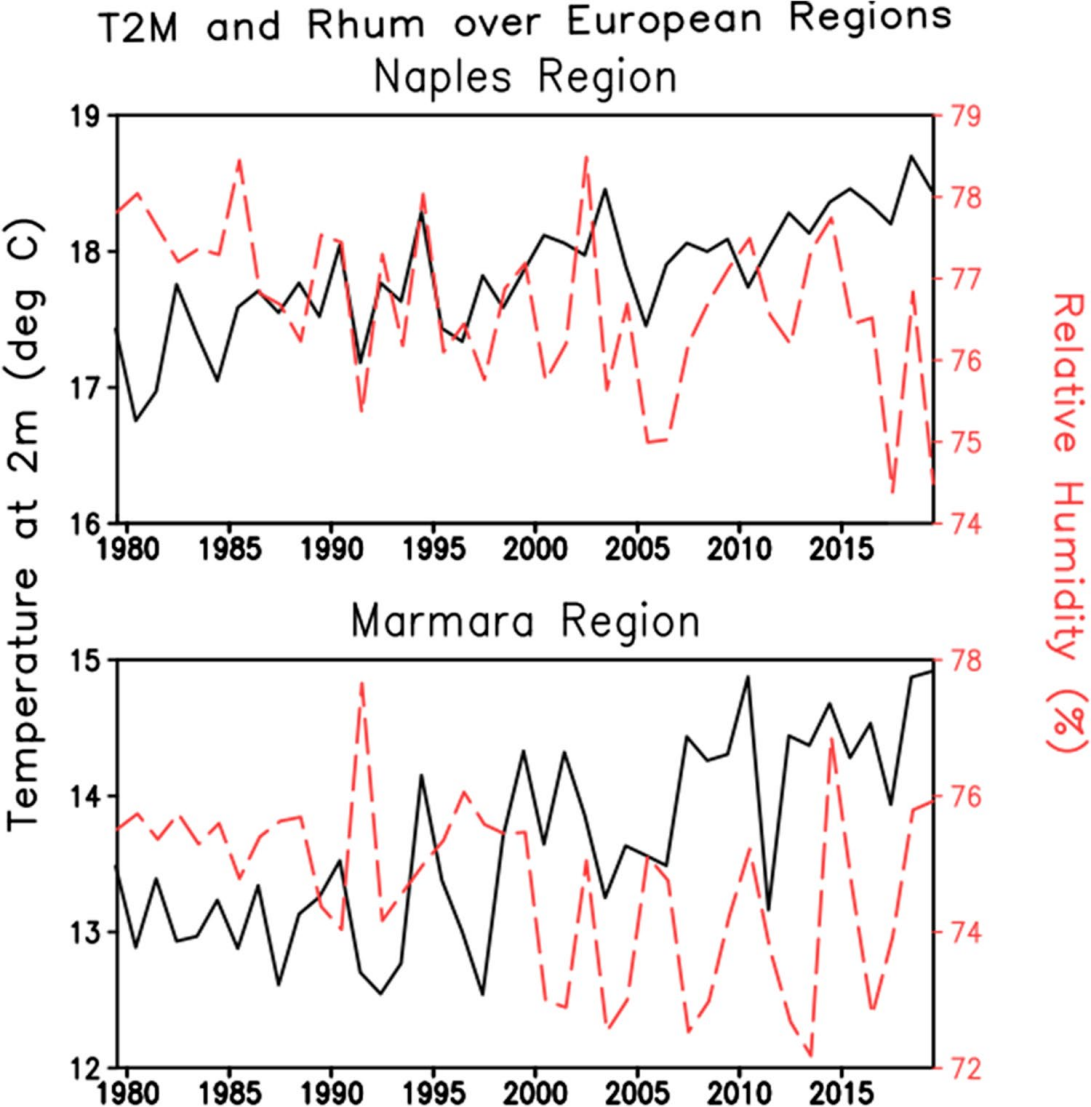
Interannual variation of temperature (°C) and relative humidity (%) over the European region for the period 1979 to 2018 from ERA5. The climate fields are averaged over Naples region (top panel) and Marmara region (bottom panel)

In Turkey, Marmara Region is the highest populated area. The HI here was distributed according to months accounted by the average values and not the extreme ones. For a period of 2007 and 2016, meteorological data like RH, wind speed, and temperature data were taken from 14 weather observation stations in Marmara Region, which were later utilized to calculate the HI (BURSALI and ŞEN 2017). For warm weather conditions (temperature greater than 21 °C), HI is calculated by the same formula given in Eq. (1). Based on these index formulas, humans live comfortably below 21 °C, semi-comfortable up till 24 °C, and very uncomfortably above the temperature of 27 °C. There is a steady increase in the temperature which is noticed in the ERA5 data (Fig. 1a). The temperature is increased by 1.5 °C (Fig. 7) and HW by 3 times (Fig. 9) as compared to the 1980s. Conversely, the relative humidity has been decreased by about 4% (Fig. 7).

#### Naples Region

Two major severe heat waves of Europe in 2003 were taken into consideration while calculating HI of Naples. The temperature is affected by the hotter African anticyclone and milder Azores anticyclone during meridional circulation (Di Cristo et al. 2007). Again, Eq. (1) is used to calculate HI when the value of air temperature and humidity are higher than 26 °C and 39%, respectively, along with the wind speed to be 2.6 m/s. The rate of the change of temperature is moreover Marmara region (Fig. 7b). It may be noticed the 2 °C warmings have already occurred as compared to the 1980s.

Table 3 depicts the health impacts faced over the wide range of HI values in the Naples and Marmara region during the summer season which are grouped. This table is also called as heat index table which quantifies the effect of HW on individuals based on their risk. This includes the continuous or prolonged physical activity of individuals at their office or any workplace. These findings may help in understanding the adaptations that can be adopted by people living in that region for the betterment of their health since it affects them adversely.

### Australia

**Fig. 8:**
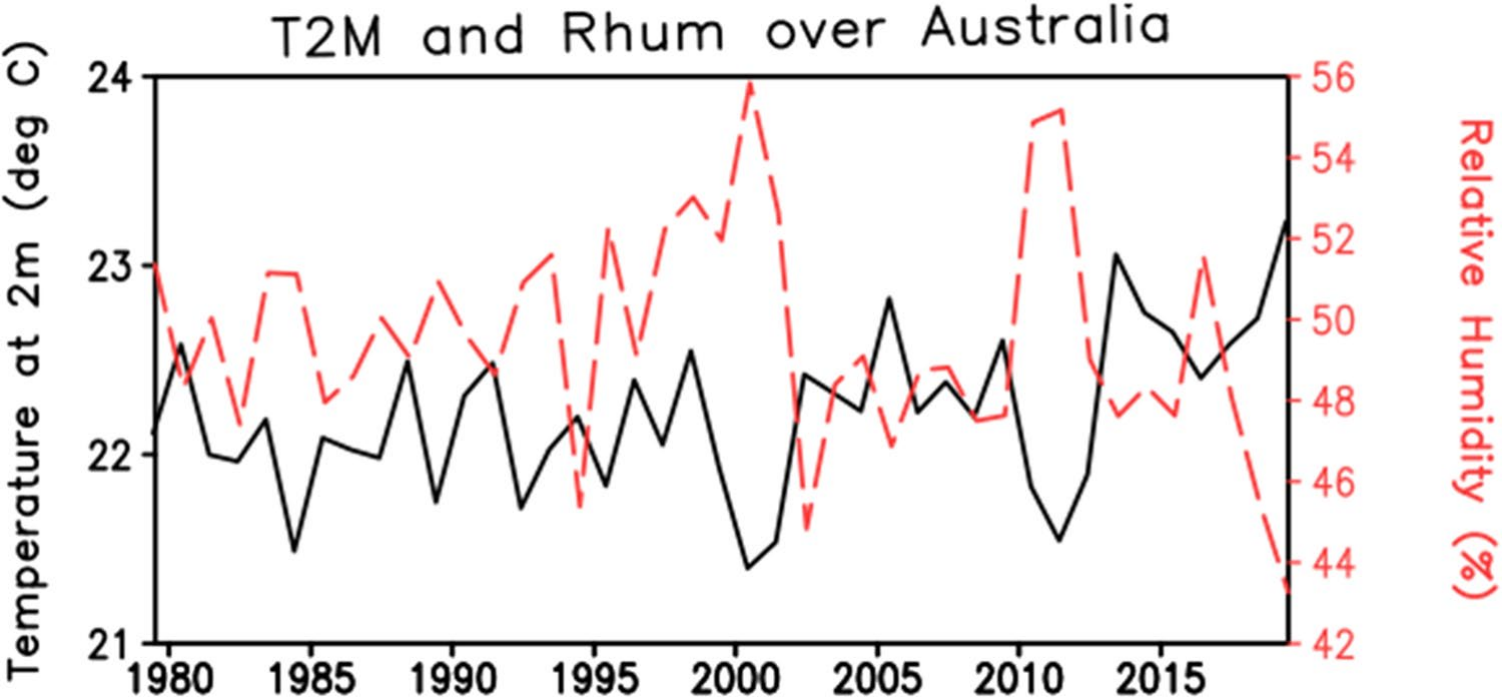
Interannual variation of temperature (°C) and relative humidity (%) over the Australian continent for the period 1979 to 2018 from ERA5. The climate fields are averaged over Australia

**Fig. 9:**
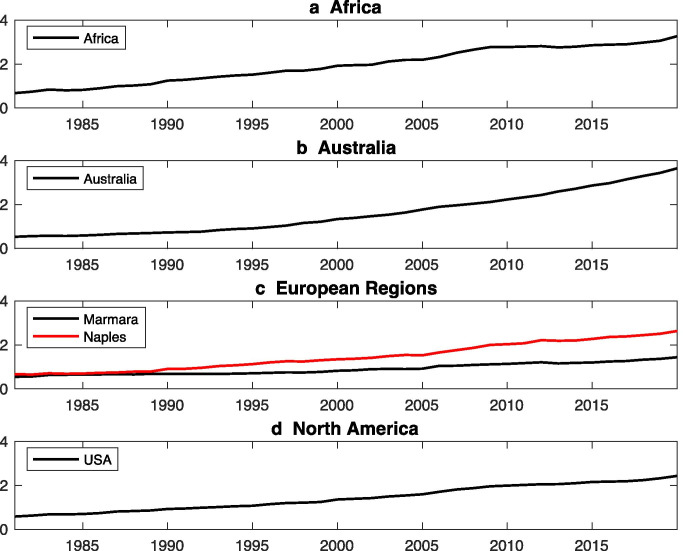
Trend of heatwave days (no. of days per year) over **a** Africa, **b** Australia, **c** Europe, and **d** North America for the period 1979 to 2018 from ERA5

Tong et al. (2010) studied the effect of extreme heat event on the health by measuring the hospital admissions in Brisbane, Australia which has sub-tropical climate (Tong et al. 2010). In this, the authors summarize the different definitions of HW used between 1996 and 2005 for Brisbane, Australia. Total ten definitions of HW were used in this study but for HI used is based on Steadman Eq. (1). Ten definitions of HW include the daily maximum temperature greater than or equal to 1 to 5% for consecutively more than 2 to 5 days. This paper indicates that it is difficult to rely upon or use one definition of HW for different regions but rely on the Steadman Eq. (1). Increase in temperature (Fig. 8) and number of heatwaves days (Fig. 9) have been found.

## Discussion

**Fig. 10:**
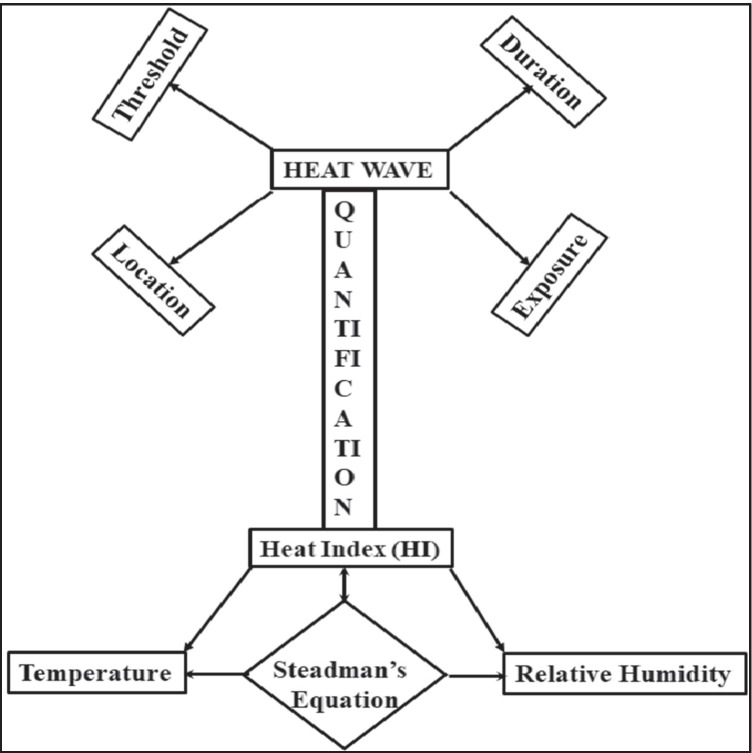
Parametric variation of heat wave and heat index

There are several diverse definitions of HW which are adapted by the researchers on the basis of their local climatic zones. These HW definitions are based on the duration of HW, exposure threshold of temperature (e.g., a relative threshold or an absolute threshold), and temperature indicator (e.g., daily average, minimum and maximum temperature), etc. as shown in Fig. 10. Since HW is the consequence of climate change, air pollution, and/or Global Warming which is quantified mathematically by using HI, therefore, the definition of HW depends upon different parameters and HI depends upon the relative humidity and temperature, which is used by Steadman in his equation by using the regression technique (Fig. 10). This Steadman equation (Eq. (1)) was derived in the 1980s and still, researchers used this equation to quantify the effect of HW.

The main purpose of this study focused on the definition of HW and HI for different countries which is characterized by the data accumulated from different sources. Overall, findings indicate that (i) at one region, HW may be termed for consecutive several days and at the other, even for 1 day. (ii) HI is consistently dependent on few parameters like air temperature and relative humidity, (iii) Steadman’s equation is still used for quantification of the effect of HW. This study helps to understand an outlook for developing/modification of the existing HI equation for a country like India where the diverse temperature is observed by either developing an assortment of such equations for the entire country with special parameters included to result into the different equations for different regions in India due to the medley of environmental factors like temperature, relative humidity, etc. Steadman’s equation was developed in the 1980s when the environmental conditions were entirely different in comparison to the present.

From Fig. 1, it is observed that temperature is high in the last decade 2009–2018 in comparison to the decade 1979–1988. The temperature rises to 3 °C during the period of 2009–2018 in comparison to the period of 1979–1988. In Fig. 1 and Figs. 3, 4, 5, 6, 7, a temperature range of 20 to 30 °C is observed which seems quite low as per the temperature given in the generic definition of heatwave, i.e., nearly 35 °C (Table 1). This is because the figures represent the annual average value that also includes winter season temperature thereby lowering the overall average annual temperature. Hence, if only the average value of the summer season is considered, especially peak intense heat days, then the value automatically reaches the heatwave range. So, the finding that there is an increase in temperature by 2–3 units will remain the same even after excluding the winter and monsoon season data. Therefore, necessary modifications in the existing equation are crucial. The alternative is to develop even-handed HI equations for different regions with similarities in their environmental condition to find the most suitable, comforting, and habitable zones. Many countries calculated HI which indicates the effect of the mortality rate of any region. The impact of HI is not limited to direct death but also other health issues like heat stroke, sunstroke, heat cramps, breathing difficulty, and decreased conscious level, and significantly lower diastolic blood pressure which makes the calculation and study of the Indian HI paramount. This paper reviewed different countries and their respective HW definition and relation between them.

The most discussed topic of present scenario in which relation of COVID1-9 cases with meteorological parameters are discussed (Awasthi et al. 2021; Singh and Vishal Mishra 2021; Tosepu et al. 2020) and many researchers proposed that COVID-19 cases increase with the increase in temperature during the summer (Bashir et al. 2020; Singh and Vishal Mishra 2021). During the summer, frequency and intensity of HW increases, who have already showed significant rise in premature death that poses high risk especially on the vulnerable groups due to Corona virus (Pijls et al. 2021). Hence it is necessary to modify the existing recommendations of heat-related illness with the consideration of infection due to different type of viruses (Reilly et al. 2021).

It is necessary and right time to identify the importance of HW and HI, as it helps in understanding the behavior of environmental conditions to live comfortably in a particular region and to reduce mortality and other diseases (Carmona et al. 2017; Shartova et al. 2018). Keeping this thing in mind; a review on the definition of HW and equations has been done because of studies done in different continents. The scrutiny of different studies in the present article has brought out the salient methods adopted by different countries to find HI relations and values in addition to heatwaves.

It is surmised from the survey that all the countries do not follow the same definition of HW as given by the World Meteorological Organization (WMO) i.e., “A period during which the daily maximum temperature exceeds for more than five consecutive days by the maximum normal temperature of 9°F (5 °C)” but most of the countries have modified the definition as per their regional metrological data. Quantitatively, HI confirmed that the temperature experienced during the summer months with high humidity values is more in comparison to the other months. Generally, the different researchers used the Steadman equation for the calculation of HI. Accordingly, different countries developed or suggested their own precautionary steps to minimize the effects of HW caused by heatstroke and heat exhaustion like living in air-conditioned homes, etc. A warning system based on HI can be designed to study the effect of extreme events based on combined observation of high daytime temperatures, warm nighttime temperature, high humidity, and light winds for several successive days.

## Conclusion

It is inferred from different studies that there is no exact definition of HW, as these vary with respective regions. Different studies implied that it is difficult to develop a generic formula for HW for all the countries worldwide. It is evident from the ERA5 data that the temperature has increased by 1–2 °C and number of heatwaves has been increased three times and in most of the continents in the last 40 years. It is observed from HI calculation that both relative humidity and temperature are the main components for the equation and in most cases; Steadman’s equation is used to quantify the effect of an extreme event like HW. Hence, to develop/modify an equation of HI depends upon a diverse range of meteorological parameters like temperature, RH, etc. Therefore, an enormous study of such parameters is required.

The development of a generic index for HW requires special correction parameters (or factors) or a value or a relation with respect to region. For this purpose, study on a mass level is required and countries need to be selected region-wise and based on meteorological variations so that every perspective of diverse variation in weather can be included. This study summarizes the basic ideas about the HW and HI so that this knowledge can provide a platform to develop or modify the existing definitions and formulas so that necessary steps can be taken to minimize the hazardous effect of extreme events like HW. As there are several definitions for HW, but for HI, Steadman’s equation is still used as a generic equation. There is a need to develop separate HI equations as per different geographical and climatic conditions. Therefore, the development/modification of the appropriate HI equation is needed for an hour and acts as future scope for this paper. The mathematical aspect of the HI equation can be handled by the mathematical expert with the joint effort of environmentalists for the development of HI.

## Data Availability

All data used in this study are freely available and can obtained directly from the source: ERA5 data (https://cds.climate.copernicus.eu/live/queue). Alternatively, the data and codes can be made available on request to the authors.

## Abbreviations

HW: Heat wave
HI: Heat index
